# Separated Electron–Phonon and Phonon–Phonon Scatterings Across Interface in Thin Film LaCoO_3_/SrTiO_3_

**DOI:** 10.1002/advs.202305900

**Published:** 2023-11-20

**Authors:** Wenjie Hao, Minghui Gu, Zhenyun Tian, Shaohua Fu, Meng Meng, Hong Zhang, Jiandong Guo, Jimin Zhao

**Affiliations:** ^1^ College of Physics Sichuan University Chengdu 610065 China; ^2^ Beijing National Laboratory for Condensed Matter Physics Institute of Physics Chinese Academy of Sciences Beijing 100190 China; ^3^ School of Physical Sciences University of Chinese Academy of Sciences Beijing 100049 China; ^4^ Songshan Lake Materials Laboratory Dongguan Guangdong 523808 China

**Keywords:** coherent acoustic phonons, electron–phonon coupling, heterostructures, phonon–phonon scattering, ultrafast spectroscopy

## Abstract

Electron–phonon coupling (EPC) and phonon–phonon scattering (PPS) are at the core of the microscopic physics mechanisms of vast quantum materials. However, to date, there are rarely reports that these two processes can be spatially separated, although they are usually temporally detached with different characteristic lifetimes. Here, by employing ultrafast spectroscopy to investigate the photo‐carrier ultrafast dynamics in a LaCoO_3_ thin film on a (100) SrTiO_3_ substrate, intriguing evidence is found that the two interactions are indeed spatially separated. The EPC mainly occurs in the thin film, whereas PPS is largely in the substrate, especially at the several atomic layers near the interface. Across‐interface penetration and decay of optical phonons into acoustic phonons thus naturally occur. An EPC strength *λ_Eg_* = 0.30 is also obtained and an acoustic phonon mode at 45.3 GHz is observed. The finding lays out a cornerstone for future quantum nano device designs.

## Graphical abstract

Electron–phonon coupling (EPC) and phonon‐phonon scattering (PPS) are at the core of the microscopic physics mechanisms of vast quantum materials. Here, evidence of spatially separated EPC and PPS is unveiled by using ultrafast spectroscopy on a 40 nm LaCoO_3_ thin films grown on SrTiO_3_ substrate. The EPC mainly occurs in thin film and the PPS occurs largely in the substrate, especially at the several atomic layers in the substrate nearby the interface.

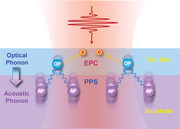

## 1 Introduction

Electron–phonon coupling (EPC) is essential in condensed matter physics, leading to pivotal phenomena such as superconductivity,^[^ ^1^, ^3^ ^‐3]^ ultrahigh thermal conductivity,^[^ ^4^ ^]^ charge density wave,^[^ ^5^ ^]^ colossal magneto‐resistant,^[^ ^6^ ^]^ etc. To date, there has been rarely reports on experimental determination of the EPC strength in transition metal perovskites, except for superconducting oxides.^[^ ^7^ ^]^ Needless to say, understanding the EPC is also important for such non‐superconducting oxides to reveal their underlying microscopic mechanisms.^[^ ^8^, ^9^ ^]^

Transition‐metal perovskite LaCoO_3_ exhibits a diamagnetic insulating low‐spin phase ($t_{2g}^{6}e_{g}^{0},\;S\;\;=\;\;0$) at low temperatures, whereas it becomes metallic at higher temperature with the Co ions assuming a higher spin state ($t_{2g}^{4}e_{g}^{2},\;S\;=\;\;2$ or $t_{2g}^{5}e_{g}^{1},\;S\;=\;\;1$).^[^ ^10^, ^11^, ^12^, ^13^ ^]^ Recently, it was found that epitaxial tensile‐strained LaCoO_3_ thin films become ferromagnetic below 85 K,^[^ ^15^, ^16^, ^17^, ^18^ ^]^ exhibiting application potentials in magnetic devices. To date, the EPC strength in LaCoO_3_ has rarely been measured, especially for thin film samples, although the EPC is one of the primary concerns.^[^ ^12^, ^15^ ^]^ Moreover, the phonon–phonon scattering (PPS) in LaCoO_3_ has rarely been investigated either.

It has rarely been reported whether the EPC and PPS are spatially separated in such oxides or other solids, although it is well known that these two processes are largely detached temporally by exhibiting different characteristic lifetimes.^[^ ^19^, ^20^ ^]^ Ultrafast time‐resolved pump‐probe spectroscopy is the most viable experimental tool to detect both the EPC and PPS in quantum materials.^[^ ^2^, ^3^, ^21^, ^22^ ^]^ In parallel, coherent phonon can also be generated and detected by ultrafast pump‐probe spectroscopy.^[^ ^23^, ^24^, ^25^ ^]^

In this work, we investigate the fluence‐dependent photo‐carriers dynamics in a 40 nm thick LaCoO_3_ film on a SrTiO_3_ substrate. Two distinct relaxation processes with lifetimes *τ*_fast_ = 0.2 ps and *τ*_slow_ = 0.9 ps are experimentally observed. The nominal EPC strength *λ_E_*_g_ is experimentally determined to be 0.30. We also detected a coherent acoustic phonon in our experiment. Significantly, we identify that the EPC and PPS are basically spatially separated by the interface of the sample.

## 2 Results

### 2.1 Photo‐Carrier Relaxation Dynamics in LaCoO_3_/SrTiO_3_

**Figure 1:**
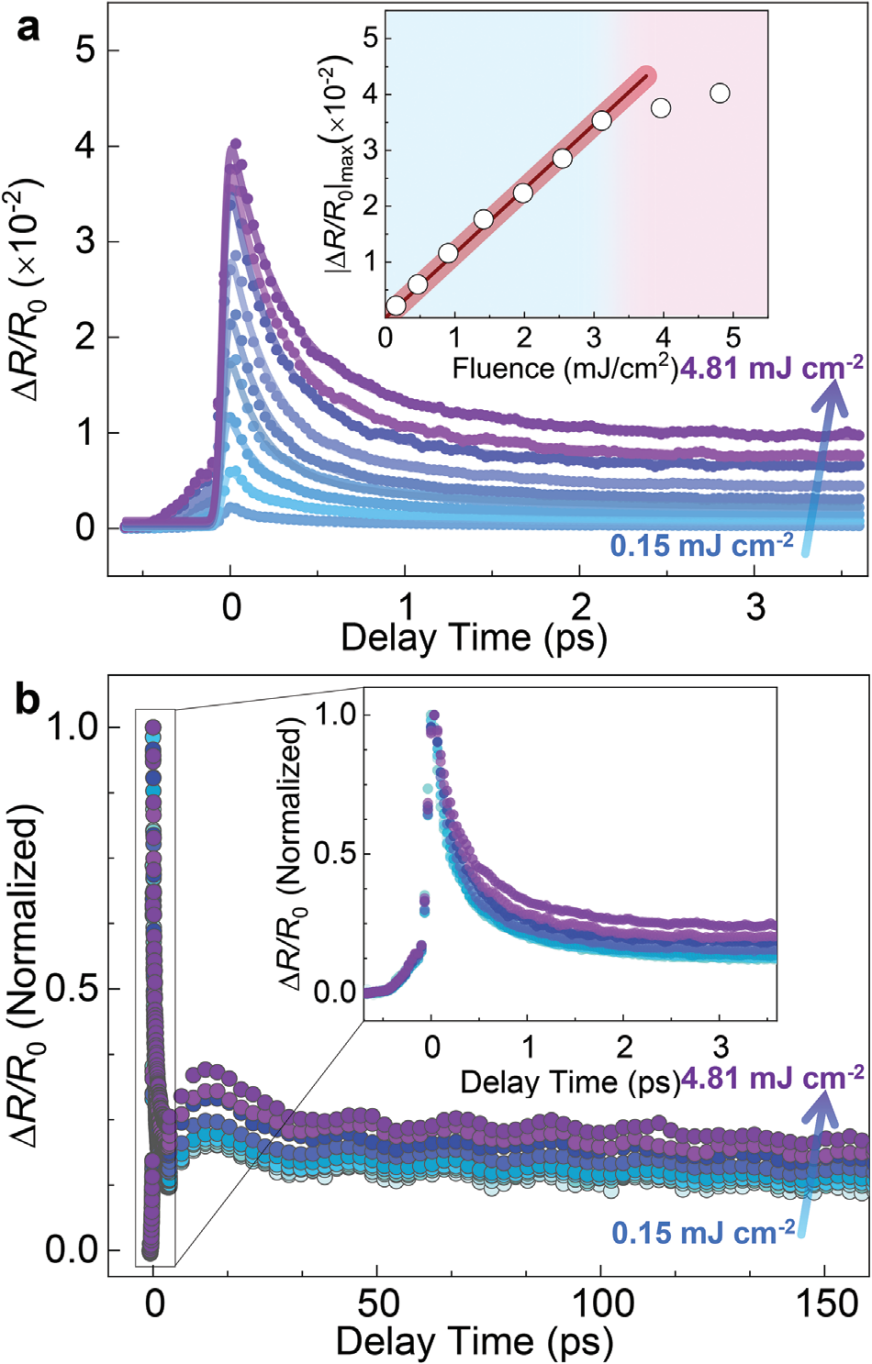
Relative transient differential reflectivity of LaCoO_3_/SrTiO_3_. a) Fluence‐dependent relative differential reflectivity Δ*R/R*_0_. Solid curves: fitting results using Equation (1). The arrow marks the increase in pump fluence. Inset: fluence‐dependence of |Δ*R*/*R*_0_|_max_. Blue and pink colors: non‐thermal and thermal effect regimes. Solid line: linear fit. b) Normalized Δ*R/R*_0_. Inset: zoom‐in view of the normalized signal Δ*R/R*_0_.

We detect the relative transient differential reflectivity Δ*R/R*_0_ of LaCoO_3_/SrTiO_3_ as a function of delay time, for which the data recorded at various pump fluences are presented in **Figure** **1**. In Figure 1a, the dots are the experimental results and the solid curves are fitting results (see a latter paragraph for a quantitative description). The signal we measure is proportional to the density of the photo‐excited carriers (abbreviated as photo‐carriers), which is intrinsically due to the Fermi transition and obeys the Fermi golden rule.^[^ ^2^, ^26^ ^]^ Upon the pump pulse excitation, the density of the photo carriers reaches a maximum value at time *t* = 0 fs, then decays through various relaxation channels, including the EPC, PPS, electron‐hole recombination, etc.^[^ ^21^ ^]^

In the inset of Figure 1a we summarize the max value of |Δ*R*/*R*_0_| as a function of the excitation fluence. The value |Δ*R*/*R*_0_|_max _ increases with fluence and a linear fit (solid line) to the signal |Δ*R*/*R*_0_|_max _ can cover most of the range of the experimental condition. At above 3.2 mJ cm^−2^ an off‐linear behavior arises, which indicates the occurrence of a thermal effect in the experiment. Note that the off‐linear (i.e., saturation) behavior in |Δ*R*/*R*_0_|_max_ is a strict criterion for identifying whether there is a thermal effect (for details, see the Supporting Information of ref. [2] and references therein). The thermal (pink) and non‐thermal (blue) effect regimes are depicted by different colors as a guide for the identification.

To better reveal the fluence dependence of the photo‐carriers ultrafast dynamics, the normalized Δ*R/R*_0_ is shown in Figure 1b. The data are normalized to the |Δ*R/R*_0_|_max_ value at the highest fluence excitation. To see the initial dynamics clearly, we illustrate a higher temporal resolution view of the scanning trace in the inset of Figure 1b. With increasing fluence, the normalized Δ*R/R*_0_ exhibits prominent changes, whereby the relaxation becomes more gradual. This reveals that the ultrafast photo‐carriers relaxation in LaCoO_3_/SrTiO_3_ is clearly dependent on the pump fluence. Note that all the data in this work are one identical set of experimental results, although they are presented in different ways to emphasize different aspects. The transient reflectivity of the SrTiO_3_ substrate is also measured, which is significantly different from that of the LaCoO_3_/SrTiO_3_ sample (Figure S2, Supporting Information). The control experiment demonstrates that the photo‐carriers relaxation dynamics in Figure 1a is mainly attributed to the LaCoO_3_ thin film.

We quantitatively analyze the experimental results. Because the electron–phonon interaction has a characteristic lifetime at the order of 1 ps,^[^ ^2^, ^4^, ^19^, ^20^ ^]^ and the presence of a hump centered at 10 ps in the dynamics^[^ ^27^ ^]^ may affect the assignment of the relaxation components, we choose the data fitting range to be from −0.7 to 3.6 ps to minimize the interference of the hump. Before we do the quantitative data fitting, we use a log‐scale coordinate to show Δ*R/R*_0_ (Figure S4, Supporting Information), which more clearly reveals how many components are present in the photo‐carriers relaxation dynamics. As seen, there are three components, and the slowest one is very flat, which can be reasonably represented by a constant term. A convoluted exponential‐decay function is employed to fit the photo‐carriers relaxation dynamics, along with a decaying cosine function to fit the coherent phonon, as:

(1) $$\begin{array}{ccc} \Delta R/R_{0} & = & \left(A_{\mathrm{fast}}e^{-t/\tau_{\mathrm{fast}}}+A_{\mathrm{slow}}e^{-t/\tau_{\mathrm{slow}}}+A_{0}\right)⊗\left(\frac{1}{\sqrt{2\pi p}}e^{t^{2}/2p^{2}}\right)\\ & & +\;A_{\mathrm{phonon}}e^{-t/\tau_{\mathrm{phonon}}}\cos\left(\Omega t+\varphi \right) \end{array}$$

where *A* and *τ* represent the amplitude and lifetime, respectively, the subscript fast, slow, and 0 mark the three components, $\frac{1}{\sqrt{2\pi p}}e^{t^{2}/2p^{2}}$is the Gaussian response function,^[^^28]^ Ω is the angular frequency of the coherent phonon, and *φ* is the initial phase of the coherent phonon. The fitting results compare well with our data [Figure 1a].

**Figure 2:**
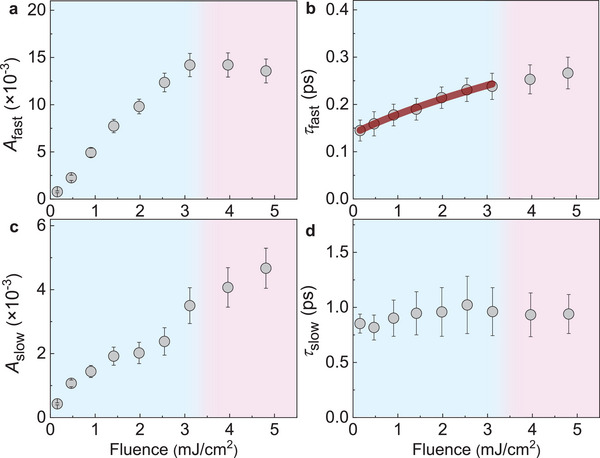
Fluence‐dependence of the amplitudes and lifetimes. Quantitatively obtained fitting results: a) *A*_fast_, b) *τ*_fast_, c) *A*_slow_, and d) *τ*_slow_. Solid curve in b): Fitted curve using a FDM^[^ ^4^ ^]^ of obtaining the EPC strength at room temperature.

The quantitative analysis of the fluence‐dependent results yields fluence‐dependent amplitudes and lifetimes, which are summarized in **Figure** **2**. The *A*_fast_ and *A*_slow_ exhibit positive correlations with the fluence in the non‐thermal effect regime [Figures 2a,c], which is in line with the results shown in the inset of Figure 1. The value of *τ*_fast_ slightly increases with fluence (Figure 2b), and *τ*_slow_ is nearly a constant (Figure 2d). Following the convention, the fast component is dominated by the EPC, and the slow component is mainly connected to the PPS. ^[^ ^2^, ^3^, ^4^ ^]^ Such assignment is based on the characteristic interaction times for different processes (Figure S5, Supporting Information),^[^ ^19^ ^]^ which is experimentally tested true and consistent in previous investigations. From the fast component, we can obtain the explicit value of the EPC strength *λ*.

### 2.2 Obtaining the EPC Strength *λ*

In a recent work,^[^^4]^ we developed a method to obtain *λ* by the fluence dependence of the fast component, for which the advantages are two‐folded: 1) one can obtain the EPC strength *λ* at room temperature, which usually does not vary much with temperature, and 2) one can circumvent the frequently encountered dilemma that the light penetration depth and the heat capacity coefficient of a material are usually unavailable. The initial work^[^ ^28^ ^]^ is developed for low temperature and medium‐high fluence regime. Later on, we have developed a more comprehensive version, which is extended to room temperature (thus easier to implement) and can also be applied to medium‐high fluence regime.^[^ ^4^ ^]^ We name this model as fluence‐dependence model (FDM).^[^ ^4^, ^28^ ^]^ The FDM is indeed derived from the Allen model.^[^ ^29^ ^]^ There have also been several other sophisticated discussions and models along this direction.^[^ ^30^, ^31^, ^32^, ^33^ ^]^ In our FDM approach, the value of *λ* is related to the fast component lifetime *τ*_fast_ by

(2) $$\tau_{\mathrm{fast}}=\frac{\pi k_{B}^{2}\sqrt{T_{L}^{2}+\Theta F}\left(\sqrt{T_{L}^{2}+\Theta F}-T_{L}\right)}{3\lambda \hbar^{2}\langle \Omega^{3}\rangle \left[\frac{1}{e^{\hbar \langle \Omega \rangle /\left(k_{B}\sqrt{T_{L}^{2}+\Theta F}\right)}-1}-\frac{1}{e^{\hbar \langle \Omega \rangle /\left(k_{B}T_{L}\right)}-1}\right]}$$

where *k_B_* is the Boltzmann constant, *T_L_* is the lattice temperature, Ω is the angular frequency of the phonon (usually optical phonon^[^ ^34^ ^]^), and Θ is an effective absorption coefficient of fluence (when a laser beam of fluence *F* is incident on the sample, the electron temperature increases from the equilibrium temperature *T_L_* to $\sqrt{T_{L}^{2}+\Theta F}$). At medium‐high fluence regime whereby the sample assumes the non‐thermal regime (i.e., the density of photocarriers is proportional to the fluence, see the inset of Figure 1a), Θ is a constant.

Using the equation of FDM, the fitting curve compares well with the experimental results at various fluences [Figure 2b]. We obtain that *λ*˂Ω^3^>/<Ω> = 294.9 ± 25.4 ps^−2^ (i.e., 129.3 ± 11.1 meV^2^). Usually, we take the lowest energy optical phonon mode as an example to obtain the nominal EPC strength for a quantum material. For our LaCoO_3_/SrTiO_3_ sample, it has four *E*_g_ modes, where the lowest energy *E*_g_ mode is the lowest energy optical phonon mode (*E*_g_) for the material. The second highest energy *E*_g_ mode is related to the orbital‐phonon coupling and the Jahn–Teller distortion.^[^ ^35^ ^]^ Still, in the Raman result, the second lowest *E*_g_ mode is much more prominent than the lowest energy *E*_g_ mode, and it is the second lowest energy optical phonon mode (another *A*_gg_ mode is silent).^[^ ^36^ ^]^ We take the second lowest *E*_g_ mode (with a frequency of 175.3 cm^−1 [^ ^35^, ^36^, ^37^ ^]^) to be the characteristic phonon mode to obtain the nominal EPC strength. In such a way, we obtain *λ_E_*_g_ = 0.30. Note that the EPC strength of LaCoO_3_/SrTiO_3_ we obtain here is close to those of superconducting oxides YBa_2_Cu_3_O_6.5_ (*λ* ≥ 0.25) and La_1.85_Sr_0.15_CuO_4_ (*λ* ≥ 0.5).^[^ ^31^ ^]^ The *λ* = 0.30 is a regular EPC value, in the middle of the various EPC values reported. Such an EPC strength is enough to cause significant Jahn–Teller distortion.^[^ ^12^, ^15^ ^]^

### 2.3 Coherent Phonon in Thin Film LaCoO_3_/SrTiO_3_

**Figure 3:**
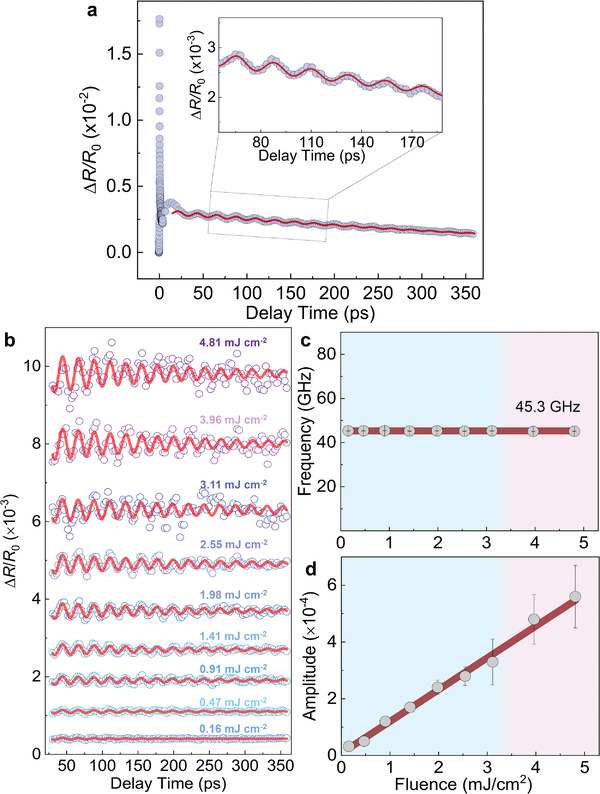
Pump fluence dependence of the coherent phonon oscillation. a) Ultrafast photo‐carriers relaxation dynamics under 1.41 mJ cm^−2^ pump fluence. Red wave: fitting curve of the coherent phonon oscillation with relaxation. b) Coherent phonon oscillation at different fluences (data are offset for clarity). Solid curves: decaying cosine functions. c,d) Fluence dependence of the phonon frequency and amplitude. Dark red lines: constant and linear fits to the data.

Furthermore, as seen in Figure 1b, there is a coherent oscillation that is unambiguously observed in our fluence‐dependent Δ*R/R*_0_ signal. We re‐plot one of the signals (with the fluence of 1.41 mJ cm^−2^) in **Figure** **3**a. Here, Figure 3a shows the data with a longer temporal range than that of Figure 1. The data at a relatively longer time scale are fitted by a red wavy curve using a decaying cosine function, which corresponds to the phonon term in Equation (1). A regular periodic oscillation is clearly seen. The inset of Figure 3a provides a closer zoom‐in view of the oscillation and the fitting curve compares well with the data. A broad hump is observed at the 4–30 ps range, which is not a part of the coherent phonon oscillation, due to the opposite phase. Previous investigations in cobalt perovskite had assigned it to the propagation of the photo‐induced metallic domain.^[^ ^38^, ^39^ ^]^ In this work we mainly focus on the coherent phonon, rather than this hump.

In Figures 3b‐d, we present the quantitative analysis of the coherent phonon. To better reveal the coherent oscillation, we subtract the photo‐carriers relaxation from the Δ*R/R*_0_ signal. The coherent oscillations at different fluences are illustrated in Figure 3b, which are offset for clarity. Each experimental oscillation trace is fitted by the decaying cosine function in Equation (1). All the values of *A*_phonon_, *τ*_phonon_, *φ*, and Ω (see Equation (1)) are obtained through the data fitting in Figure 3b. The phonon frequency Ω is shown in Figure 3c, whose average value is 45.3 GHz, nearly unchanged even up to the high fluence regime whereby the thermal effect inaugurates. The value of the phonon frequency is much smaller than a regular optical phonon frequency. We attribute it to be a coherent acoustic phonon, which is generated by the transient thermal strain induced by the ultrafast light pulses.^[^ ^24^, ^40^, ^41^ ^]^ Here the phonon frequency is independent of the film thickness (Figure S8, Supporting Information).^[^ ^24^, ^42^ ^]^ The phonon amplitudes *A*_phonon_ are summarized in Figure 3d, which increase linearly with fluence. Thus, unlike that for the photo‐carriers dynamics (Figure 1a and Figure 2a), no prominent saturation on coherent phonons is observed in the thermal effect regime. This indicates the lattice does not experience irreversible damage by shining the ultrafast light pulses on. Similar property has also been found in other materials (e.g., in Cd_3_As_2_^[^ ^25^ ^]^).

**Figure 4:**
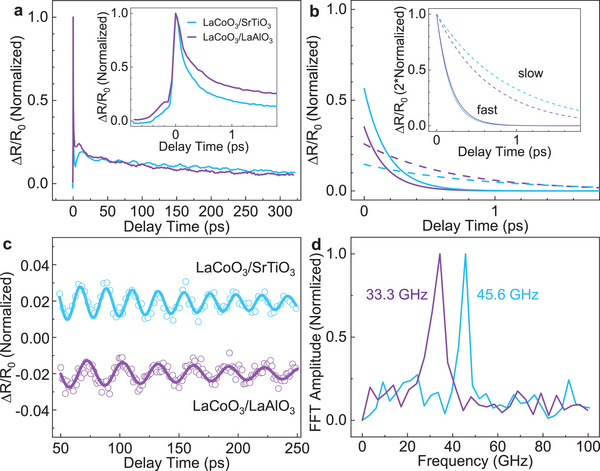
Photo‐carriers relaxation dynamics of LaCoO_3_/SrTiO_3_ and LaCoO_3_/LaAlO_3_. a) Normalized differential reflectivity Δ*R/R*_0_ under close pump and probe fluences. Inset: zoom‐in view for initial temporal range. b) The fitting components for fast and slow components in the inset of 5a. Inset: Normalized components. c) Coherent phonon oscillation with cosine decay fitting (offset for clarity). d) Fourier transformation of the oscillations. Note, for LaCoO_3_/SrTiO_3_, the pump fluence is 0.91 mJ cm^−2^ and the probe fluence is 0.13 mJ cm^−2^.

Significantly, we investigate the effect of the substrate on the photo‐carriers relaxation dynamics by comparing the ultrafast dynamics of two different samples. In the control experiment, the second sample is a 40 nm thickness LaCoO_3_ on a (100) LaAlO_3_ substrate. The pump and probe beam fluences are 0.86 and 0.14 mJ cm^−2^, respectively. The dynamics we obtain is shown in **Figure** **4**a, which is normalized to compare with that of the LaCoO_3_/SrTiO_3_ sample. The data for LaCoO_3_/SrTiO_3_ are obtained under a pump fluence of 0.91 mJ cm^−2^ and a probe fluence of 0.13 mJ cm^−2^, which are nearly identical to those for the LaCoO_3_/LaAlO_3_ sample. In the longer temporal range, the dynamics of these two samples exhibit an apparent difference. However, for the shorter temporal range, the dynamics for the two samples nearly overlap in the initial range (see the inset of Figure 4a for better revealed the ultrafast relaxation at initial stage). This indicates that the fast and slow components behave in a different way. To better illaustrate the data, following the aforementioned procedures (Equation (1)), we display the fast and slow components for both samples in Figure 4b, along with a normalized version in its inset. The solid curves are fast components and the dashed curves are slow components. The fast components for the two samples are nearly overlapped; as a contrast, the slow components for the two samples are clearly different. While the lifetime *τ*_fast_ = 0.17 ± 0.03 ps is nearly identical for both samples (*τ*_fast_ for the LaCoO_3_/SrTiO_3_ sample is 0.18 ps (Figure 2b), the lifetime *τ*_slow_ = 0.67 ± 0.18 ps is different from that for the other sample (*τ*_slow_ = 0.90 ± 0.17 ps (Figure 2d) for the LaCoO_3_/SrTiO_3_ sample). These results indicate that the EPC strength for the two samples is very close; however, the PPS rate in LaCoO_3_/SrTiO_3_ is noticeably lower than in LaCoO_3_/LaAlO_3_.

In the control experiment, we follow the same procedure to analyze the coherent acoustic phonon in the LaCoO_3_/LaAlO_3_ sample. The oscillations were obtained by subtracting the photo‐carriers relaxation in Figure 4a, and are presented in Figure 4c, both fitted with cosine decaying functions. The frequency domain results are obtained through Fast Fourier Transformation (FFT) (see Figure 4d). Interestingly, the two frequency domain peaks are located at 33.3 (for LaCoO_3_/LaAlO_3_) and 45.6 GHz (for LaCoO_3_/SrTiO_3_), respectively. While these two values are different, we find that the value for LaCoO_3_/LaAlO_3_ is in well agreement with that in Fe_2_O_3_/LaAlO_3_,^[^ ^43^ ^]^ and that for LaCoO_3_/SrTiO_3_ is in agreement with that in Fe_2_O_3_/SrTiO_3_ and LaRhO_3_/SrTiO_3_.^[^ ^42^, ^43^ ^]^

## 3 Discussion

We summarize a few typical reported coherent acoustic phonon frequencies in oxides thin films, as well as in some semiconductor thin films, in **Table** **1**. All these values are obtained at room temperature and probed with 800 nm light pulses. From the table, the coherent acoustic phonon frequency is nearly identical for samples with identical substrates, regardless of the material of the thin films. This indicates that the coherent acoustic phonons are all mainly generated in the substrate. Note that for Figure 3b by sample we mean the whole heterostructure including the film and substrate. All these results are in consensus with each other, indicating that the coherent acoustic phonons are mainly determined by the substrate. It is very plausible that the coherent acoustic phonons are generated (especially at a few atomic layers in the substrate nearby the interface) and detected in the substrates (Figure S9, Supporting Information). The penetration depth of LaCoO_3_ is reported to be 110 nm,^[^ ^44^ ^]^ which allows for the prominent transmission through a 40 nm thick thin film. In such a scenario, the pump pulse generates a transient thermal strain, producing longitudinal temperature gradients that spread at the interface between the film and substrate to generate the coherent acoustic phonon.^[^ ^40^, ^45^ ^]^ Note that the coherent acoustic phonon in a bare SrTiO_3_ substrate is not easy to observe.^[^ ^42^ ^]^ Usually, one needs the interference between the reflections from the surface and the strain wave to detect the coherent acoustic phonon (Figure S9, Supporting Information).^[^ ^42^ ^]^

**Table 1:** Coherent acoustic phonon frequencies in various oxide and semiconductor thin film samples (from this work and adapted from^[^ ^42^, ^43^, ^46^, ^47^ ^]^).

Sample	Phonon frequency [GHz]
LaRhO_3_/SrTiO_3_(110)	45^[^ ^42^ ^]^
LaCoO_3_/SrTiO_3_(100)	45.3^[this work]^
Fe_2_O_3_/SrTiO_3_(100)	44.7^[^ ^43^ ^]^
Fe_2_O_3_/BaTiO_3_(100)	30.9^[^ ^43^ ^]^
LaCoO_3_/LaAlO_3_(100)	33.3^[this work]^
Fe_2_O_3_/LaAlO_3_(100)	33.9^[^ ^43^ ^]^
LaRhO_3_/(LaAlO_3_)_0.3_(Sr_2_TaAlO_6_)_0.7_(110)	35.05^[^ ^42^ ^]^
GaSb/GaAs	43^[^ ^46^ ^]^
Co_2_MnAl/GaAs	43.5^[^ ^47^ ^]^

The phonon frequencies in samples with identical substrates exhibit close values, not depending on the thin film material. Identical thin films on different substrates exhibit different frequency values. These facts indicate that the acoustic phonons are dominated by the substrates.

**Figure 5:**
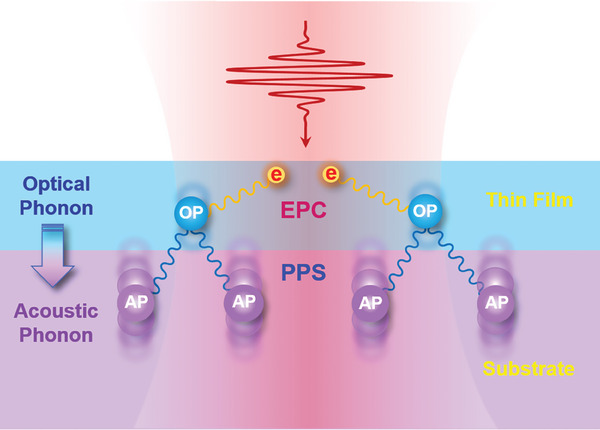
Schematic illustration of the separated EPC and PPS processes across the interface. The vibrational balls depict the real‐space atomic position fluctuations. OP: optical phonons; AP: acoustic phonons. The pump laser pulses instantly generate excited state photo‐carriers (within femtoseconds), which then excite optical phonons mainly within the thin film (with a typical interaction lifetime of 0.15–0.25 ps, see Figure 2). Concomitantly, these optical phonons annihilate to create acoustic phonons mainly within the substrate, especially at the several atomic layers in the substrate nearby the interface (with a characteristic interaction lifetime of 0.8–1.2 ps, see Figure 2). Such a process naturally involves the penetration of phonons across the interface.

We schematically illustrate the scenario underlying the whole process in **Figure** **5**. The photo‐carriers are excited to the excited states and then relax through the coupling with optical phonons in the thin films (not mainly in the substrate).^[^ ^4^, ^34^ ^]^ Energy is exchanged between the photo‐carriers and the thin film crystal lattice. Consequently, the thin film lattice absorbs the energy from the photo‐carriers, generating a vast quantity of non‐equilibrium optical phonons, which relax mainly through PPS, decaying into lower energy acoustic phonons. The deviation in the PPS rate in LaCoO_3_/SrTiO_3_ and LaCoO_3_/LaAlO_3_ suggests that this OP→AP relaxation process mainly occurs in the substrate, especially at the several atomic layers in the substrate nearby the interface, instead of the thin film. Note that the inset of Figure 4b shows a very similar EPC relaxation rate, indicating that strain^[^ ^48^ ^]^ does not affect EPC (Figure S6, Supporting Information); also, possible effects caused by interfacial structural configuration or band alignment^[^ ^49^, ^50^ ^]^ will be masked by our LaCoO_3_ 40 nm thick film, thus becoming negligible (Figure S7, Supporting Information).

Assuming that it is other than the above scenario—supposing the EPC and PPS occur both in the thin film or substrate, we should observe nearly identical *τ*_fast_ and *τ*_slow_ in the control experiment, which is apparently not the fact. Hence, we conclude that the EPC and PPS are spatially separated by the interface. This scenario is also confirmed by the distinctive coherent acoustic phonons observed in samples with distinctive substrates. Owing to such a scenario, the OP→AP decaying process inevitably penetrates the interface, which is in line with the longitudinal nature of the acoustic phonons.^[^ ^24^, ^25^ ^]^ As a result, the penetration and annihilation/creation of phonons perpendicular to the interface^[^ ^3^, ^51^, ^52^, ^53^ ^]^ naturally occur. A temporal evolution for the ultrafast processes is depicted in the caption.

## 4 Summary

In summary, we investigate the ultrafast dynamics of LaCoO_3_/SrTiO_3_ and perform the control experiment with a LaCoO_3_/LaAlO_3_ sample. By the lifetime of the fast photo‐carriers relaxation component, we obtain the EPC strength in the LaCoO_3_ thin film to be *λ*˂Ω^3^>/<Ω> = 129.3 ± 11.1 meV^2^, which corresponds to a nominal EPC strength of *λ_E_*_g_  = 0.30. A coherent acoustic phonon mode with a frequency of 45.3 GHz is also generated and detected. We attribute it to light pulse‐induced thermal strain. Intriguingly, through the control experiment, we discover that the EPC mainly occurs in the thin film and the PPS is dominated by the substrate, especially at the several atomic layers in the substrate nearby the interface, whereby the optical phonons penetrate across the interface to decay into acoustic phonons. Our findings reveal a rarely observed/reported phenomena that can hardly be detected by any other experimental means, and lays down an important physics mechanism foundation for the relevant future designs of quantum nano devices.

## 5 Experimental Section

Ultrafast laser pulses with 800 nm central wavelength, 70 fs pulse duration, and 250 kHz repetition rate were used. The spot diameters of the pump and probe beams were 60 and 55 µm, respectively, on the sample surface. The pump fluence ranges from 0.16 to 4.81 mJ cm^−2^, while the probe fluence was kept at 0.13 mJ cm^−2^. The fluences *F* were obtained through *F*= 4*W*/*R_r_* π*d* ^2^, where *W* was the laser beam power, *R_r_* was the repetition rate of the laser, and *d* was the beam spot diameter. Cross‐polarization detection was implemented in order to reduce noise. We conducted all the experiments at room temperature (i.e., 298 K).

Our samples were grown by using the pulsed laser deposition method, where two ≈100 unit‐cell‐thick (approximately 40 nm) LaCoO_3_ films were grown on twin‐polished SrTiO_3_(100) and LaAlO_3_(100) substrates, respectively. The oxygen partial pressure was optimized at 15 Pa and the growth temperature was 670 °C. After in‐situ annealing for 1 h, the samples were cooled down to room temperature under the same oxygen pressure (15 Pa). The substrates were 5 × 5 × 0.5 mm^3^ in volume. The general grown method, X‐ray diffraction, and reflection high‐energy electron diffraction characterizations were presented in Supporting Information, Figure S1. Our magnetic and electrical characterizations shown that there was no prominent oxygen vacancy (or in‐gap state) in the SrTiO_3_(100) and LaAlO_3_(100) substrates (Figure S3 Supporting Information S1).

## Conflict of Interest

The authors declare no conflict of interest.

## Author Contributions

J.Z. conceived the idea and supervised the project. W.H. and J.Z. conducted the ultrafast spectroscopy experiment. M.G., M.M., and J.G. fabricated the sample and did the characterization. W.H., Z.T., S.F., and J.Z. analyzed the data. With constructive inputs from H.Z. and J.G., W.H. prepared the draft and J.Z. wrote the paper.

## Supporting information



Supporting Information

## Data Availability

The data that support the findings of this study are available from the corresponding author upon reasonable request.
