# Supplementary material for: Separated Electron–Phonon and Phonon–Phonon Scatterings Across Interface in Thin Film LaCoO3/SrTiO3
Source: Adv Sci (Weinh). 2023 Nov 20;11(2):2305900. doi: 10.1002/advs.202305900 (PMC10787100; doi:10.1002/advs.202305900)
Supplement: Supplementary file 1 — Supporting Information [file ADVS-11-2305900-s001.pdf]

ADVANCED SCIENCE
Open Access

Supporting Information

for *Adv. Sci.*, DOI 10.1002/advs.202305900

Separated Electron–Phonon and Phonon–Phonon Scatterings Across Interface in Thin Film $LaCoO_3/SrTiO_3$

Wenjie Hao, Minghui Gu, Zhenyun Tian, Shaohua Fu, Meng Meng, Hong Zhang, Jiandong Guo* and Jimin Zhao**

Supporting Information

Separated electron-phonon and phonon-phonon scatterings across interface in thin film $LaCoO_3/SrTiO_3$

Wenjie Hao,[1,2] Minghui Gu,[2,3] Zhenyun Tian,[2] Shaohua Fu,[2] Meng Meng,[2,3] Hong Zhang,[1,*] Jiandong Guo,[2,3,*] and Jimin Zhao[2,3,4,*]

[1] *College of Physics, Sichuan University, Chengdu 610065, China*

[2] *Beijing National Laboratory for Condensed Matter Physics, Institute of Physics, Chinese Academy of Sciences, Beijing 100190, China*

[3] *School of Physical Sciences, University of Chinese Academy of Sciences, Beijing 100049, China*

[4] *Songshan Lake Materials Laboratory, Dongguan, Guangdong 523808, China*

* Corresponding authors: jmzhao@iphy.ac.cn; jdguo@iphy.ac.cn; hongzhang@scu.edu.cn

Sample preparation and XRD and RHEED characterizations

The 40 nm (*i.e.*, 100 unit cell layers) thick $LaCoO_3$ films are grown on 2-sides polished (100) $SrTiO_3$ and (100) $LaAlO_3$ substrates by using pulsed the laser deposition method. The KrF excimer laser (248 nm of wavelength) fluence and repetition rate are set as 1.2 J/cm^2 and 2 Hz, respectively. The oxygen partial pressure is optimized at 15 Pa and the growth temperature is 670 °C. After in-situ annealing for 1 hour, the samples are cooled down to room temperature under the same oxygen pressure (15 Pa).

The X-ray diffraction (XRD) measurement results and reflection high-energy electron diffraction patterns of the two samples are shown in Fig. S1. The characterization results illustrate high crystalline qualities of the $LaCoO_3/SrTiO_3$ and $LaCoO_3/LaAlO_3$ samples.

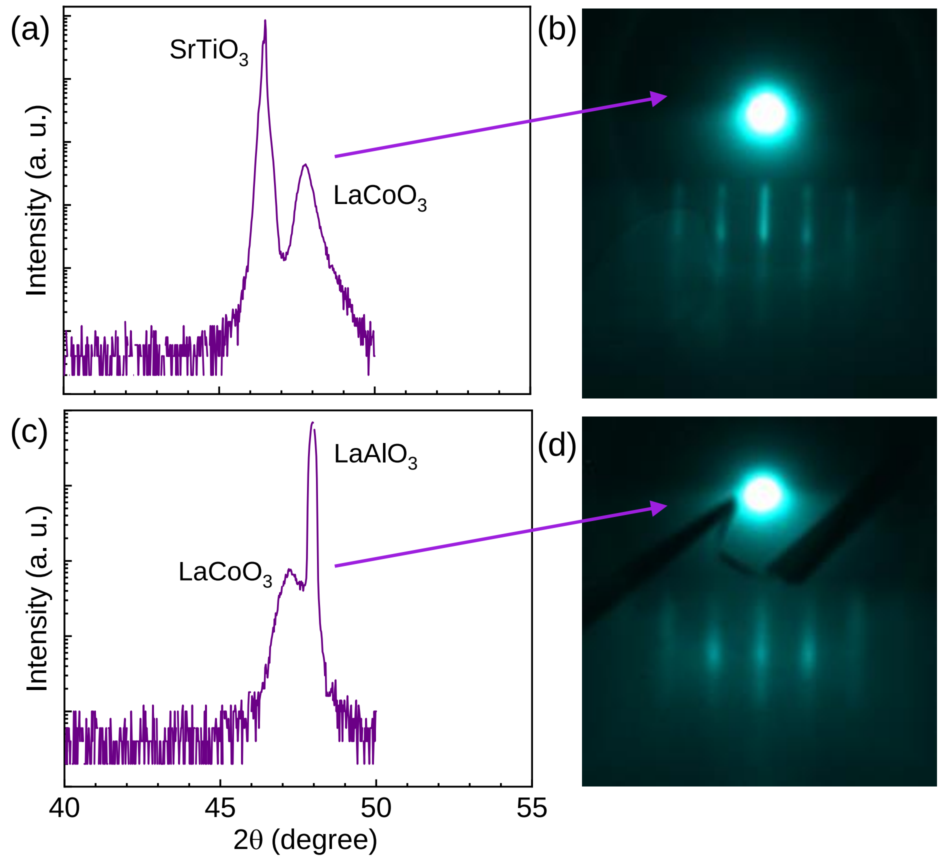

FIG. S1 Characterization of the $LaCoO_3/SrTiO_3$ and $LaCoO_3/LaAlO_3$ samples. (a, c) The (002) XRD peaks, and (b, d) Reflection high-energy electron diffraction patterns for the $LaCoO_3/SrTiO_3$ and $LaCoO_3/LaAlO_3$ samples, respectively.

Control experiment on the photo-carrier relaxation dynamics: $LaCoO_3/SrTiO_3$ sample vs $SrTiO_3$ substrate

To verify whether the photo-carriers relaxation dynamics originates from the $LaCoO_3$ film or substrate, we carry out a control experiment by measuring the transient differential reflectivity of the bare $SrTiO_3$ substrate. The normalized data are presented in Fig. S2 (red curve). The pump and probe beam fluences are 0.7 and 0.2 mJ/cm^2, respectively, which is quite similar to the experimental condition for the $LaCoO_3/SrTiO_3$ sample. The pump and probe beam fluences for the $LaCoO_3/SrTiO_3$ sample are 0.91 and 0.13 mJ/cm^2, respectively. The result of the latter is illustrated by blue curve in Fig. S2. It can be seen the ultrafast photo-carrier relaxation dynamic for $SrTiO_3$ is distinctly different from that of the $LaCoO_3/SrTiO_3$ sample, which suggests that the transient reflectivity signals of the $LaCoO_3/SrTiO_3$ sample are mainly from the $LaCoO_3$ thin film.

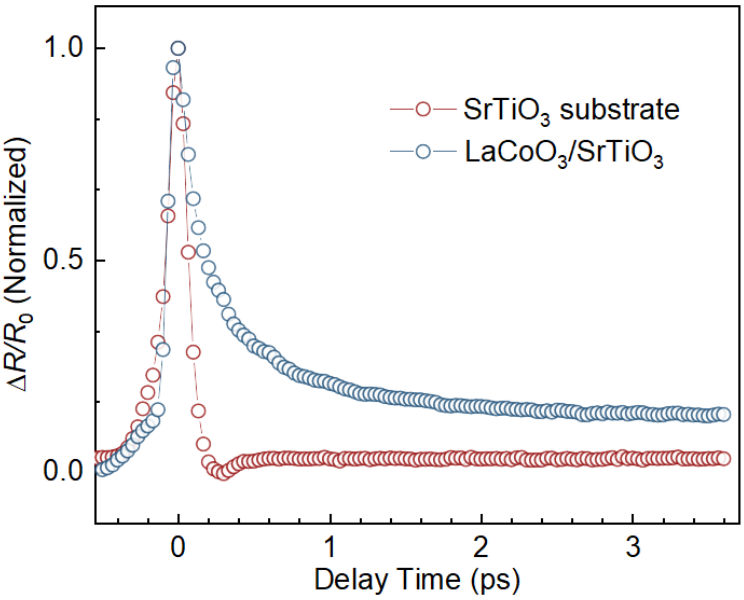

FIG. S2. Normalized photo-carriers relaxation dynamics for $LaCoO_3/SrTiO_3$ (blue curve) and $SrTiO_3$ substrate (red curve).

No prominent oxygen vacancy (*i.e.*, in-gap state)

In this work, we performed a control experiment (see Fig. S2) using bare $SrTiO_3$ without oxygen vacancy. And the LCO/STO heterostructure studied in this work was

grown under oxygen pressure of 15 Pa. Normally such a relatively high oxygen pressure results in low density of oxygen vacancies (V_os) in the film. Furthermore, the low V_os density in the LCO/STO sample can be verified by direct characterizations: 1) The magnetism. It has been demonstrated that the LCO films exhibits tensile strain-induced ferromagnetism and the T_c is shifted to lower temperatures with increased content of oxygen vacancy [S1]. The magnetic transition temperature T_c of the LCO/STO sample in the current work is determined as 85 K (Fig. S3(a)), indicating the low V_os density. On the other hand, considering the formation energy of V_os in LCO is significantly lower than in STO, the V_os should be distributed with a lower density in the STO substrate than in the LCO film in the LCO/STO heterostructure. Therefore, we conclude that the LCO/STO heterostructure is stoichiometric without oxygen vacancy. 2) The resistivity. It is well-known that the oxygen vacancies can lead to higher conductivity of STO. However, our heterostructure is highly insulating [Fig. S3(b)], which again demonstrates that our LCO/STO sample is almost free of V_os. 3) It is also worth mentioning that, from ultrafast spectroscopy, the EPC relaxation rates in $LaCoO_3/SrTiO_3$ and $LaCoO_3/LaAlO_3$ are nearly the same. Considering the two heterostructures have different substrate (or in-gap states associated with V_os), it can be interpreted that there is no prominent oxygen vacancy in the substrates. Otherwise, we will observe the difference.

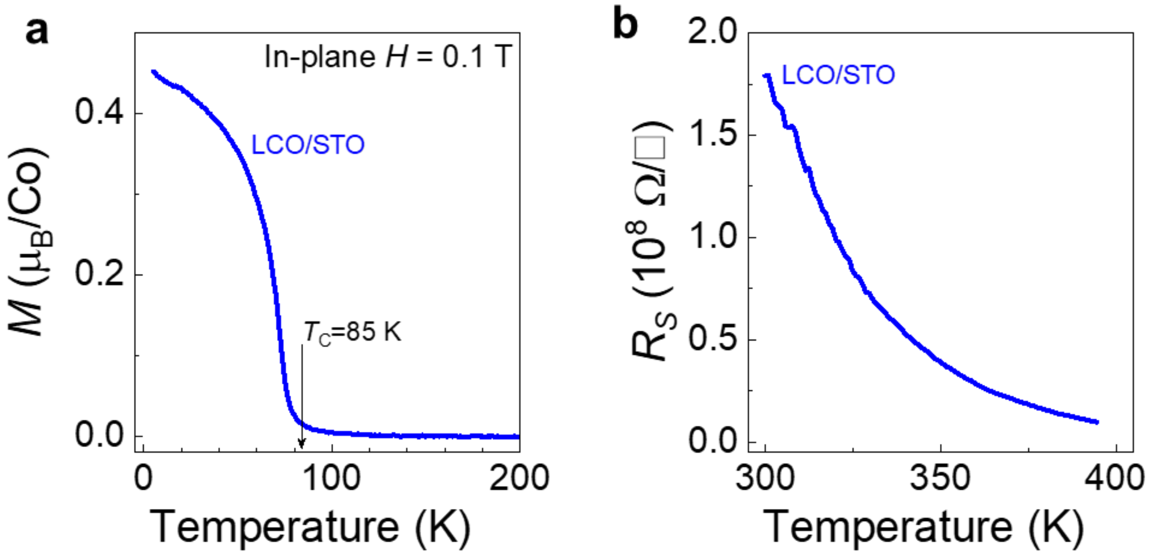

Fig. S3. (a) Magnetism moment and (b) resistivity of $LaCoO_3/SrTiO_3$.

Identifying the ultrafast relaxation components

To clearly identify the number of ultrafast relaxation components in the photo-carrier dynamic in $LaCoO_3/SrTiO_3$, we show a logarithm scale plot for a typical scanning data of $\Delta R/R_0$ taken for a fluence of 2.55 mJ/cm^2 [see Fig. S4(a)]. Three components (solid lines) are identified, with relatively clear distinction from each other. The third component (presented by a purple line) is nearly flat and we regard it as a constant. To show that treating the third component as a constant is reasonable, we employ a convoluted exponential decay function $\Delta R/R_0 = (A_{fast} e^{-t/\tau_{fast}} + A_{slow} e^{-t/\tau_{slow}} + A_0) \otimes (\frac{1}{\sqrt{2\pi p}} e^{t^2/2p^2})$ to fit the data. The fitting result is shown in Fig. S4(b) (red curve), which compares well with the experimental data. Explicitly, the three components are also illustrated, where the light blue curve is the fast component, the deep blue curve denotes the slow component, and the pink line represents the constant slowest component. Thus, we qualitatively show the 3-component analysis is reasonable. In the main text, a more rigorous procedure is used to analyze all the experimental data, namely the coherent phonon oscillations are also strictly considered, as reflected in Eq. (1).

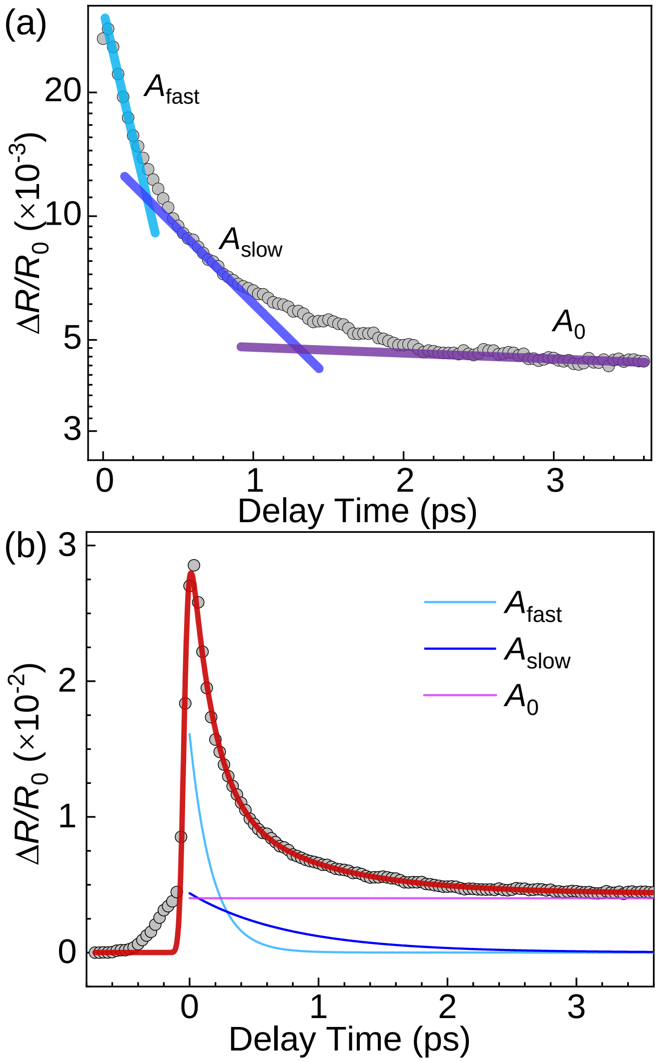

FIG. S4 (a) A logarithm-scale plot of the photo-carrier ultrafast dynamics for $LaCoO_3/SrTiO_3$ with a 2.55 mJ/cm^2 pump fluence. Solid lines: guides to the eyes to reveal the three relaxation components. (b) Time domain decomposition of the ultrafast photo-carrier dynamics. Red curve: summation of the three components as compared with the experimental data. Light blue curve: exponential decay function as a fit to the fast component. Deep blue curve: fitting curve for the slow component. Pink line: fitting line for the constant slowest component.

Slow component and phonon-phonon scattering

The time scale of the lifetime is a major factor in assigning the components. In this work, τ_{slow} is ~ 1 ps, and there is already a 0.2 ps fast component. Usually, the fast component corresponds to the e-phonon coupling (EPC) and the slow component corresponds to the phonon-phonon scattering (PPS). For the different time scales for

different processes, please refer to Ref. [S2] or Fig. 2 of Ref. [S3]. Such assignments are tested in many experiments and consistency is generally acquired for all solids. Because different materials have different exact values, the time scale may not be so strict and fluctuating range of values are often encountered.

In Fig. S5, we show that, if we merge the fast and slow components together, the curve will not be consistent with the data, yielding a poor data fitting. Therefore, it is valid that we assign two components rather than one, besides the constant term. Thus, we obtain both the fast and slow components.

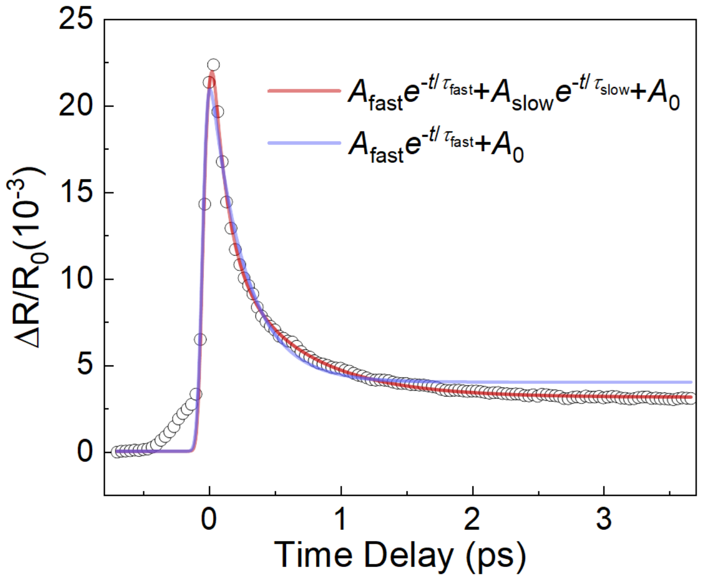

Fig. S5. Three-component fitting result (red curve) as compared with two-component fitting result (blue curve) for the $\Delta R/R_0$ signal of our $LaCoO_3/SrTiO_3$ at 1.98 mJ/cm^2.

For PPS, it can be OP (optic phonon)→OP+OP, OP→OP+AP (acoustic phonon), and OP→AP+AP. The generated OP will continue the process further. Ultimately, it will turn into OP→AP+AP. For examples, see the series Ref. [S4]. There are individual examples for OP→OP+AP [S5], OP→OP+OP and OP→AP+AP [S6, S7, and references therein]. All of these processes are seen as PPS. In our experiment, the PPS has a relatively short lifetime. Thus, we interpret OP→OP+AP has a larger portion in PPS. Note that here for ultrafast spectroscopy, when we mention PPS, we do not include the process of AP→AP+AP, or the propagation of AP, because this process is closely related to the thermal and heating effects, which has a much longer lifetime.

The effect of lattice strain on EPC and PPS

In a solid, the ultrafast relaxation is composed of several decaying channels: the e-phonon interaction (about 0.2~3 ps), PPS (about 1~500 ps), thermal diffusion (about 0.4 ns~20 μs), *etc*. In the inset of Fig. 4(b), the fast components of the two samples exhibit very similar EPC relaxation rate (the solid curves). The two samples are $LaCoO_3/SrTiO_3$ and $LaCoO_3/LaAlO_3$: the films are identical and the substrates are different. If the EPC mainly occurs in the substrate, the two fast relaxation rates cannot be the same. Hence, the inset of Fig. 4(b) clearly demonstrates that the EPC mainly occurs in the $LaCoO_3$ film. Because they are nearly identical, it also clearly indicates that strain in the $LaCoO_3$ film has negligible effect on the EPC. In solids, the electrons are much lighter than the lattice ions. Considering that Coulomb interaction dominates the e-phonon and phonon-phonon scatterings, the EPC is more sensitive than the PPS to be affected. If the strain has an effect here, it is better reflected in the EPC. Given the fact that in our sample the EPC is not affected by the strain, strain will unlikely affect the PPS either.

In a parallel consideration, for our sample, the film is 10000 times thinner than the substrate. There is no mechanism that prevents the carriers in the $LaCoO_3$ film to relax by the film or substrate. Both contributes to the relaxation and apparently the substrate contributes more (Fig. S6). The fact that the fast components have similar time constant is indeed surprising, which indicates that the EPC mainly occurs in the film. This is probably because the e-phonon interaction is very fast. The PPS has longer lifetime, thus more likely within the substrate, especially at the beginning layers near the interface.

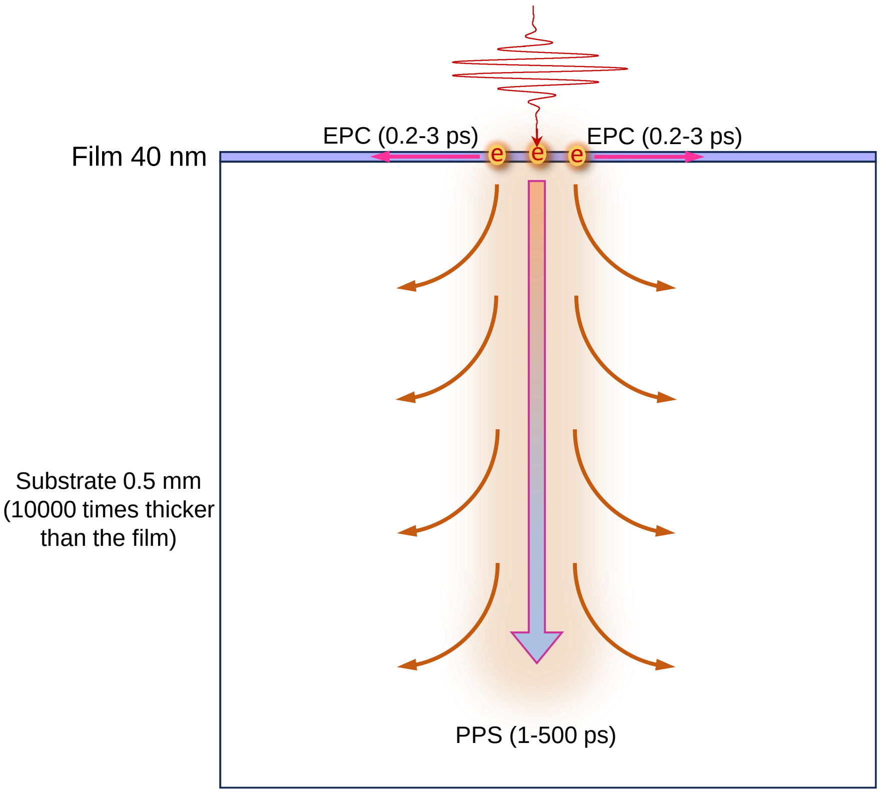

Fig. S6. Schematic of the EPC and PPS interactions in the longitudinal direction. The substrate is 10000 times thicker than the film. Thus, the substrate potentially dominates the PPS interactions.

Interfacial structural configuration and band alignment

The interfacial structural re-configuration or band alignment may potentially affect the EPC and PPS processes, too. However, the change due to a few atomic layers can be negligible compared with the thick substrate and relatively thick $LaCoO_3$ film. The signal of such interface reconfiguration or band renormalization will be mostly masked by the signals from film and substrate. For example, in Fig. S1 of Ref. [S8] (replot as Fig. S7), after no more than 10 UC of covering film, the signal from the interface is completely un-discernable in the pump-probe spectroscopy; here we have a 40 nm-thick film, which is much thicker.

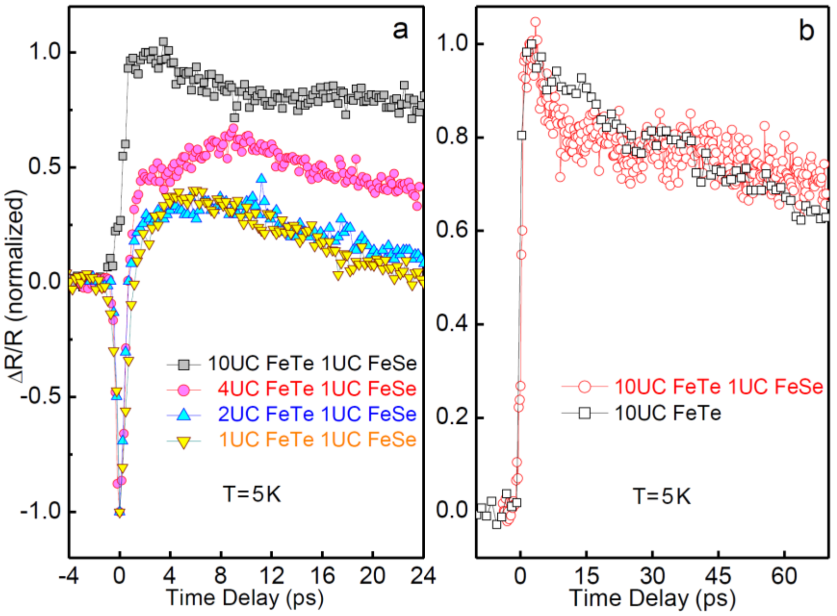

Fig. S7. Adapted from Ref. S8. Effect of the number of capping layers. When the capping layers is more than 4 UC (*e.g.*, about 10 UC), the feature of the interface lattice renormalization and band renormalization has been "erased" or "masked" to the extent of un-discernable.

Frequency of the coherent acoustic phonon (CAP): linearly depending on film thickness vs being constant

When the film is thin, standing waves of the acoustic phonon are formed, thus the frequency linearly depends on the sample thickness. However, when the sample is relatively thick (the critical thickness depends the material), there is no standing wave and the frequency of the acoustic phonon does not rely on the film thickness, thus becoming a constant (see Fig. S8, adapted from Ref. [S9], see the part with larger number of layers in the blue box). In the thick film case, the mechanism is thermal strain induced by ultrafast laser pulses (see Refs. [S9, S10]). The mechanism is such that the reflection of the probe laser beam from the surface interferes with that reflected by the thermal strain wave (see Fig. S9, adapted from Refs. [S11, S12]), thus sensing the acoustic phonon wave [S10].

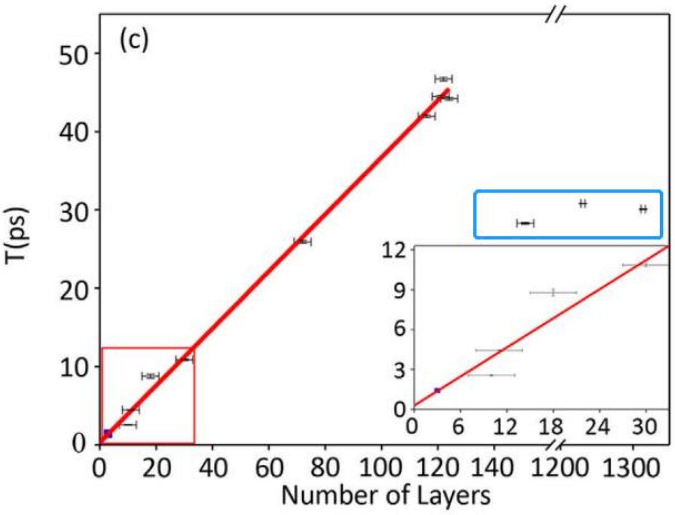

Fig. S8: Dependence of the period/frequency of the acoustic phonon on the sample thickness in MoS_2. Adapted from Ref. [S9]. Left part: linear regime. Right part: constant regime (blue box).

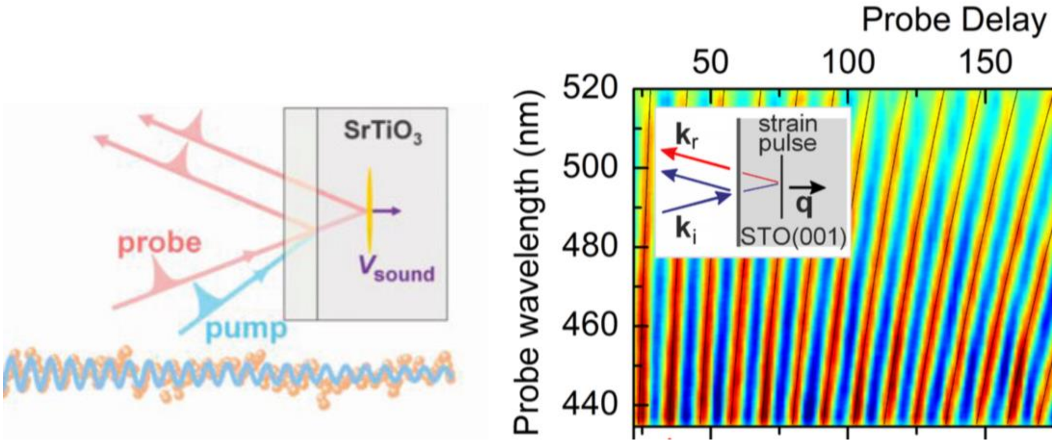

Fig. S9. Schematic illustration of the acoustic wave detection by recording the interference of the reflections from the sample surface and propagating thermal strain (adapted from Refs. [S11, S12]).

In our recent work Ref. [11] (see Fig. S10, adapted from Fig. S3 of Ref. S11), the coherent acoustic phonon exhibits a constant frequency (45 GHz) for three different samples, of which the thickness ranges from 17 to 136 nm. The material is $LaRhO_3/SrTiO_3$, which is similar to our sample here $LaCoO_3/SrTiO_3$. The film thickness of our sample is 40 nm, within the 17-136 nm range. Hence, it is very reasonable that our sample is within the constant frequency regime.

The CAP is mainly generated at the several atomic layers in the substrate near the interface.

The CAP is different from the acoustic phonons generated through PPS (i.e., by OP→AP). Thus, one might think the CAP is generated in the thin film and then it propagates into the substrate by penetrating the interface. However, this is not very true. Our film is 40 nm thick. If we estimate, the velocity of the sound wave is 5.7 nm/ps [S13]. It can be estimated that propagating from the front layer of the thin film to interface takes ~7.5 ps. If we look at our previous experimental result [Fig. S3 of Ref. S11, replotted below as Fig. S10], we find that the starting point where CAP appears is exactly correlated with the film thickness. Particularly, the time it takes before the CAP is generated is exactly the time needed for a laser-induced thermal strain pulse to propagate (with the velocity of sound wave) from the front layer atoms of the film to the interface (in the below figure, for the 17 nm, 68 nm, and 136 nm $LaRhO_3$ thin film, evident kinks can be seen at 12.5 ps, 22.5 ps, and 36 ps, respectively (marked with green arrows, note that the time zero is at t = 9 ps). These results indicate an identical sound velocity of 4.9 nm/ps in $LaRhO_3$, which is a reasonable value, considering that in $LaCoO_3$ it is 5.7 nm/ps [S13]). The several layers of atoms in the substrate at the interface is thus stimulated by the laser-induced thermal strain pulse, leading to the generation of CAP.

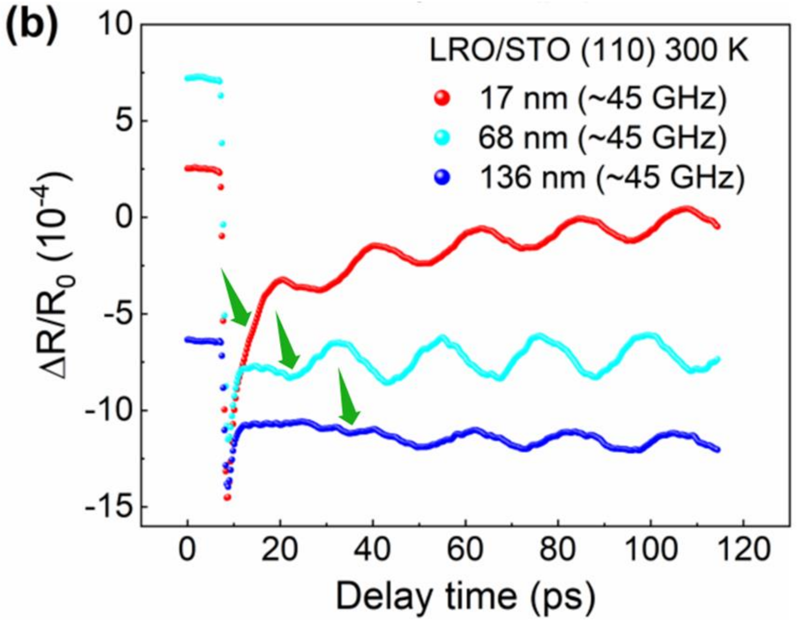

Fig. S10: Adapted from Fig. S3 of Ref. [S11]. The three $LaRhO_3/SrTiO_3$ heterostructures are all pumped and probed with 800 nm light pulses at 300 K, which is identical with our case here. Green arrows: the time when the CAP is generated.

Mechanism of OP→AP penetration across the interface

An OP in the film can generate APs in the substrate—through the interface. For example, if we take the A_g mode OP for instance, the atoms within the film vibrate perpendicularly to the surface. At the interface, such vibrations will create bumping between the film and substrate atoms. This will create vibrations of the atoms in the substrate (especially for the several layers at the interface), too, in a way seen as acoustic phonons. In such a way, the OP generated in the film decays into APs that is sustained in the substrate (Fig. S11). In Fig. S11, in the film, atoms vibrating under the OP mode will inevitably collide with atoms in the substrate, which generates APs. Note that phonons extend in a relatively large region—much larger than the atom-to-atom distance—due to the atomic collisions. Thus, this OP→AP process does not necessarily require that OP propagates into the substrate. In other words, the PPS process can occur without a prominent propagation of OP. Consequently, the EPC is mainly within the film and the PPS is mainly at the interface—the OP→AP process penetrates the interface naturally—thus the EPC and PPS are spatially separated.

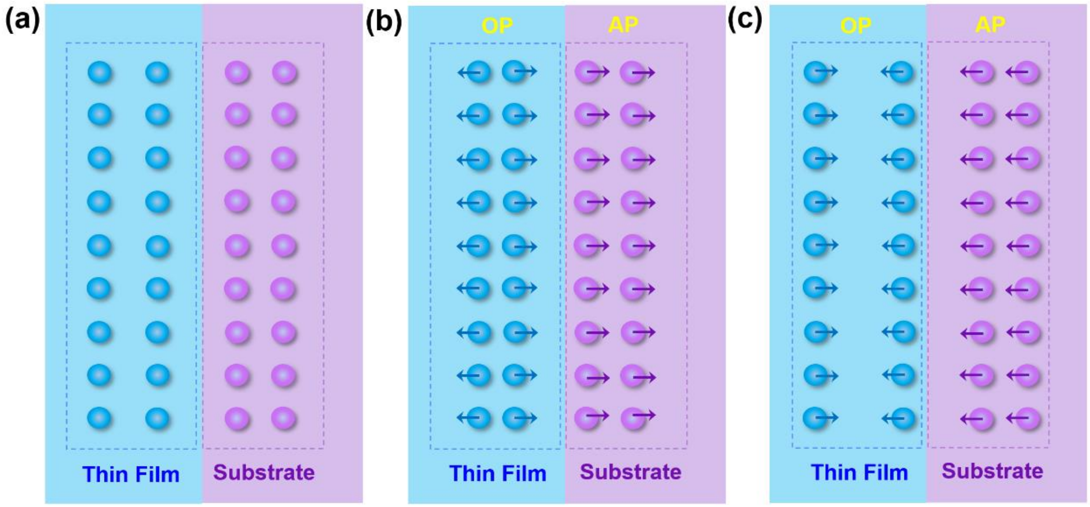

Fig. S11. Schematic of the OP→AP phonon-phonon scattering and penetration of the OP→AP process across an interface. The balls represent lattice atoms. Arrows represent

collective motions of the atoms.

The optical phonon is dispersionless at the gamma point and thus does not propagate in a single crystal. However, at an interface between two materials, the atoms in the thin film will collide with the atoms in the substrate (Fig. S11). Hence, phonon penetration [S14, S15] or phonon-phonon scattering can easily occur at the interface. On the other hand, although OPs usually have very small dispersions at gamma point, it does not mean that all the dispersions are absolutely zero (e.g., see Fig. 3 of S16, replotted as Fig. S12): there are quite many optical phonon branches with none-zero dispersions at the gamma point.

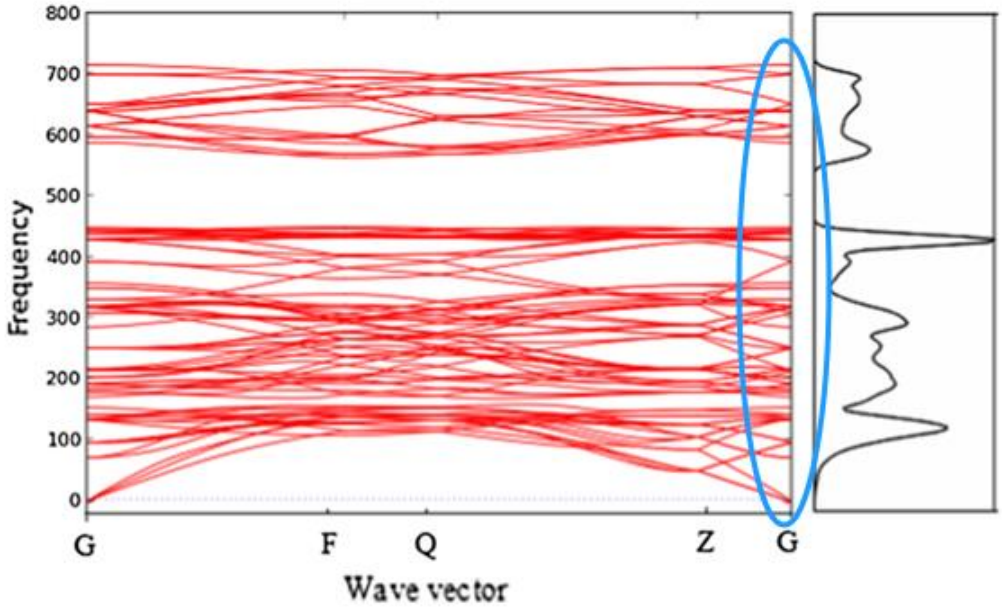

Fig. 12. Adapted from Fig. 3 of Ref. S14. The phonon dispersion spectrum (left panel) of $LaCoO_3$. The light-blue ellipse encircles quite a few non-zero dispersion branches of the phonons at the Gamma point.

Reference

[S1] D. C. Meng, H. L. Guo, Z. Z. Cui, C. Ma, J. Zhao, J. B. Lu, H. Xu, Z. C. Wang, X. Hu, Z. P. Fu, R. R. Peng, J. H. Guo, X. F. Zhai, G. J. Brown, R. Knize, and Y. L. Lu, *Strain-induced high-temperature perovskite ferromagnetic insulator*, PNAS **2018**, 115, 2873.

[S2] J. Shah, Ultrafast Spectroscopy of Semiconductors and Semiconductor Nanostructures (Springer-Verlag, Berlin, 1996)

[S3] L. Perfetti, P. A. Loukakos, M. Lisowski, U. Bovensiepen, M. Wolf, H. Berger, S. Biermann, and A. Georges, *Femtosecond dynamics of electronic states in the Mott insulator 1T-TaS_2 by time resolved photoelectron spectroscopy*, New J. Phys, **2008**, 10, 053019.

[S4] Manual Cardona et. al, *Light Scattering in Solids*, Springer

[S5] J. Chen, J. B. Khurgin, and R. Merlin, *Stimulated-emission-induced enhancement of the decay rate of longitudinal optical phonons in III–V semiconductors*, Appl. Phys. Lett. **2002**, 80, 2901.

[S6] A. V. Bragas, C. Aku-Leh, S. Costantino, Alka Ingale, J. M Zhao, and R. Merlin, *Ultrafast optical generation of coherent phonons in $CdTe_{1-x}Se_x$ quantum dots*, Phys. Rev. B **2004,** 69, 205306 (2004).

[S7] C. Aku-Leh, J. M. Zhao, R. Merlin, J. Menéndez, and M. Cardona, *Long-lived optical phonons in ZnO studied with impulsive stimulated Raman scattering*, Phys. Rev. B **2005,** 71, 205211.

[S8] Y. C. Tian, W. H. Zhang, F. S. Li, Y. L. Wu, Q. Wu, F. Sun, G. Y. Zhou, L. L. Wang, X. C. Ma, Q. K. Xue, and Jimin Zhao, *Ultrafast Dynamics Evidence of High Temperature superconductivity in single unit cell FeSe on $SrTiO_3$*, Phys. Rev. Lett. **2016**, 116, 107001.

[S9] S. F. Ge, X. F. Liu, X. F. Qiao, Q. S. Wang, Z. Xu, J. Qiu, P. H. Tan, Jimin Zhao, and D. Sun, *Coherent longitudinal acoustic phonon approaching THz frequency in multilayer Molybdenum Disulphide*, Sci. Rep. **2014**, 4, 5722.

[S10] C. Thomsen, H. T. Grahn, H. J. Maris, and J. Tauc, *Surface generation and detection of phonons by picosecond light pulses*, Phys. Rev. B **1986**, 34, 4129.

[S11] T. Sun, C. Zhou, H. L. Guo, Z. Meng, X. Y. Liu, Z. Wang, H. Zhou, Y. M. Fei, K. Qiu, F. P. Zhang, B. L. Li, X. T. Zhu, F. Yang, J. M. Zhao, J. D. Guo, J. Zhao, and Z. G. Sheng, *Coherent Phonon-Induced Gigahertz Optical Birefringence and Its Manipulation in $SrTiO_3$*, Adv. Sci. **2023**, 10, 202205707.

[S12] S. Brivio, D. Polli, A. Crespi, R. Osellame, G. Cerullo, and R. Bertacco, *Observation of anomalous acoustic phonon dispersion in $SrTiO_3$ by broadband stimulated Brillouin scattering*, Appl. Phys. Lett. **2001**, 98, 211907.

[S13] M. Zahradník, M. Kiaba, S. Espinoza, M. Rebarz, J. Andreasson, O. Caha, F. Abadizaman, D. Munzar, and A. Dubroka, *Photoinduced insulator-to-metal transition and coherent acoustic phonon propagation in $LaCoO_3$ thin films explored by femtosecond pump-probe ellipsometry*, Phys. Rev. B **2022**, 105, 235113.

[S14] S.Y. Zhang, J. Q. Guan, X, Jia, B, Liu, W. H. Wang, F. S. Li, L.L. Wang, X. C.

Ma, Q. K. Xue, J. D. Zhang, E. W. Plummer, X. T. Zhu, and J. D. Guo, *Role of $SrTiO_3$ phonon penetrating into thin FeSe films in the enhancement of superconductivity*, Phys. Rev. B **2016**, 94, 081116.

[S15] S. Y. Zhang, J. Q. Guan, Y. Wang, T. Berlijn, S. Johnston, X. Jia, B. Liu, Q.Zhu, Q.C. An, S. W. Xue, Y. W. Cao, F. Yang, W. H. Wang, J. D. Zhang, E. W. Plummer, X. T. Zhu, and J. D. Guo, *Lattice dynamics of ultrathin FeSe films on $SrTiO_3$*, Phys. Rev. B **2018**, 97, 035408.

[S16] X. Wang, Y. Han, X. J. Song, W. H. Liu, and H. Z. Cui, *Phonon spectrum and thermodynamic properties of $LaCoO_3$ based on first-principles theory*, Com. Mater. Sci. **2017**, 136, 191.
